# Phytotherapeutics: The Emerging Role of Intestinal and Hepatocellular Transporters in Drug Interactions with Botanical Supplements

**DOI:** 10.3390/molecules22101699

**Published:** 2017-10-21

**Authors:** Ghulam Murtaza, Naveed Ullah, Farah Mukhtar, Shamyla Nawazish, Saiqa Muneer, Mariam

**Affiliations:** 1 Department of Pharmacy, COMSATS Institute of Information Technology, Abbottabad 22060, Pakistan; gmdogar356@gmail.com; 2 Department of Pharmacy, University of Swabi, Swabi 23340, Pakistan; naveedjia@yahoo.com; 3 Department of Microbiology, Sardar Bahdur Khan Women University, Quetta 87300, Pakistan; fmukhtar3@gmail.coms; 4 Department of Environmental Sciences, COMSATS Institute of Information Technology, Abbottabad 22060, Pakistan; shamyla@ciit.net.pk; 5 Department of Pharmacy, University of Lahore, Lahore 54000, Pakistan; saiqamunir77@gmail.com; 6 Department of Biotechnology, COMSATS Institute of Information Technology, Abbottabad 22060, Pakistan

**Keywords:** botanical supplements, metabolism, drug transporters, P-glycoproteins, organic anion transporting polypeptides

## Abstract

In herbalism, botanical supplements are commonly believed to be safe remedies, however, botanical supplements and dietary ingredients interact with transport and metabolic processes, affecting drug disposition. Although a large number of studies have described that botanical supplements interfere with drug metabolism, the mode of their interaction with drug transport processes is not well described. Such interactions may result in serious undesired effects and changed drug efficacy, therefore, some studies on interaction between botanical supplement ingredients and drug transporters such as P-gp and OATPs are described here, suggesting that the interaction between botanical supplements and the drug transporters is clinically significant.

## 1. Introduction

Health improvement and the treatment or prevention of diseases through the use of botanical supplements has been increasingly adopted over recent decades [1]. This trend may be attributed to the general concept that botanicals are nontoxic because of their natural source and an extensive traditional usage history [2]. Besides, the general public has growing knowledge of health and easy access to botanical supplements [3]. Although, botanicals have been proven efficacious, several botanical extracts may also have noxious fractions [2,3]. On concomitant administration with drugs, botanical supplements may modulate drug metabolism or/and interact with transporters, resulting in their interaction with drugs [4]. This may subsequently influence the pharmacokinetic and pharmacodynamic properties of drugs, leading to their changed efficacy and undesired effects [5,6]. The objective of this article is to concisely discuss the interactions of botanicals and food components with transporters, with special emphasis on clinical relevance of such interactions. While a wide array of studies on in vitro mechanistic and clinical evaluation of numerous botanicals is available in literature, limited studies are described here.

## 2. Oral Absorption of Coadministered Botanical Ingredients and Drugs

The gastrointestinal tract is the site of food digestion and absorption of digestion products. A large number of studies have reported the molecular mechanisms of the absorption of lipids [7], amino acids [8,9] and sugars [10,11]. The portal vein carries the absorbed components to the liver, where hepatocytes take up or allow them pass on into the systemic circulation. The molecular transport systems involved in nutrient uptake into the liver are well documented [12]. Apart from transporters involved in nutrient uptake, the intestine and the liver cells express other transporters that mediate the uptake of xenobiotics, for example flavonoids [13,14,15].

**Figure 1:**
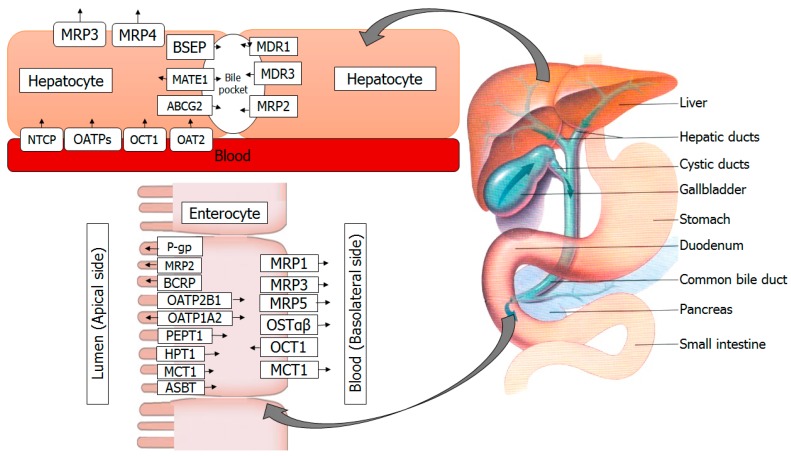
A schematic representation of transporters in human intestine and liver for movement of drugs, botanical supplements and bile salts. Note: P-gp, P-glycoprotein; OATP, organic anion transporting polypeptide; BSEP, bile salt export pump; ABC, ATP-binding cassette; ASBT, apical sodium-dependent bile acid transporter; OST, organic solute transporter; MRP, multidrug resistance-associated protein; MDR, multidrug resistance protein; NTCP, sodium taurocholate cotransporting polypeptide; MATE: multidrug and toxin extruder; OCT, organic cation transporter; OAT, organic anion transporter; MCT, monocarboxylase transporter; HPT, human intestinal peptide-associated transporter; PEPT, peptide transporter.

Xenobiotic transporters not only uptake, but also export the compounds from the enterocytes and the hepatocytes [16,17] (Figure 1). The transport function of these transporters is well studied, but their mode of interaction with and transport of botanical products is still unclear due to various reasons, such as the researchers are still unable to efficiently purify and/or detect many botanical supplements in vitro. Besides, the legislative regulation for botanical supplements is not as strict as for drug usage. Botanical supplements are legislatively categorized as medicinal products and dietary supplements in Europe and the United States, respectively [18]. In Europe, the marketing of botanical supplements is permitted on the basis of bibliographic data on their safety and efficacy profiles, while no such data is required in the United States if botanical supplements contain new food constituents.

## 3. Transporters in the Intestine

The intestinal movement of nutrients and xenobiotics takes place in accordance with the solute transport principle [19]: Facilitated diffusion is the mechanism for solute uptake (transfer from lumen into cytoplasm of epithelial cells) in the apical membrane of enterocytes, while organic anion transporting polypeptides (OATPs), also known as solute carrier organic anion transporters (*SLCO*), mediate xenobiotic uptake. OATP2B1 transporter, also abbreviated as *SLCO2B1*, is an important example of such mediators [20]. This transporter-mediated movement takes place along concentration gradient of solute across epithelial membrane. In addition, there are some transporters, known as secondary active transporters that acquire energy from the sodium or proton gradient and mediate substrate movement against concentration gradient across the apical membrane of enterocytes. These transporters can be exemplified by peptide transporter 1 (PEPT1), also termed as *SLC15A1* [21,22]. Facilitated diffusion is also involved in export of substances from enterocytes across the basolateral membrane, for example, efflux of d-glucose [23]; however, very limited information on solute carrier transporter (*SLC*) expression at the basolateral membrane is available in literature. Otherwise, botanical supplements may be effluxed actively by ATP-binding cassette (ABC) transporters present at the basolateral membrane of enterocytes, for instance, ABCC3 (also termed as multidrug resistance-associated protein 3 or MRP3) that could be involved in baicalin transport across the basolateral membrane of enterocytes [24,25].

It is remarkable that the entry of xenobiotics into the body is restricted by the efflux proteins present in the apical membrane of enterocytes. The examples of these efflux proteins that pump out the xenobiotics into gut lumen are ABCB1 [multidrug resistance protein (MDR1) or P-glycoprotein (P-gp)], ABCC2 (MRP2) and ABCG2 [26,27]. This efflux system can actively limit the transfer of drugs and botanical supplements to the systemic circulation, providing protection against noxious substances [28]. Other substrates for apical ABC transporters include the metabolic products of various xenobiotics such as botanical supplements. The metabolic products may be glucuronides [29].

## 4. Transporters in the Liver

The intestinal membrane acts as a transport-limiting barrier for xenobiotics, i.e., xenobiotics gain access to portal circulation, and thus to liver, after passing the intestinal obstacle. Then the liver acts as a second transport limiting barrier for xenobiotics before getting access to systemic circulation. The fenestrated sinusoidal endothelial cell layer separates the portal blood from the basolateral membrane of hepatocytes, revealing the direct contact of this basolateral membrane with the portal blood plasma [30]. Several transporters including OATP1B1 (*SLCO1B1*), OATP1B3 (*SLCO1B3*), OATP2B1, organic cation transporter 1 (OCT1) (*SLC22A1*), OCT3 (*SLC22A3*), organic anion transporter 2 (OAT2) (*SLC22A7*) and OAT7) (*SLC22A9*) are expressed on the basolateral membrane of hepatocytes [17,31], while hepatocytes are expected to have additional transporters. ABC transporters such as P-gp, ABCG2 and MRP2 expressed at the canalicular membrane mediate the extrusion of xenobiotics and their metabolites from hepatocytes into bile [32]. Bile transports these xenobiotics into the intestine, from where these molecules are carried back to liver via portal blood, constituting entreohepatic recycling [33]. The canalicular membrane not only contains ABC transporters but also expresses some other transporters such as the SLC transporter multidrug and toxin extruder 1 (MATE1) (*SLC47A1*), showing its probable contribution to xenobiotic extrusion into the canaliculus [34]. Hepatocyte may also push back the xenobiotics and their metabolites into the portal blood plasma. This transport process is facilitated by ABC transporters such as MRP3 and MRP4 (*ABCC4*) [32].

The liver not only handles the xenobiotics but also synthesizes bile that transports bile salts from the sinusoidal blood plasma into the canaliculi. Sodium taurocholate cotransporting polypeptide (NTCP) (*SLC10A1*) and OATPs mediate the hepatocellular uptake of bile salts, while the bile salt export pump (BSEP) (*ABCB11*) mediate the extrusion of bile salts from hepatocytes into the calaiculi [35]. Xenobiotics may interfere with bile salt extrusion from hepatocytes into the canaliculi, leading to the elevated levels of intracellular bile salt that may result in the development of cholestasis [36].

## 5. Botanical Supplement-Mediated Cellular Uptake and Efflux

In contrast to chemically synthesized compounds, botanical supplements are impure products due to their procurement as extracts containing multiple substances. Moreover, the compositions of botanical products are not consistent due to the use of different extraction methods. In addition, these products may exhibit significant batch-to-batch variation in the quantities of the individual components. Therefore, the in vivo outcomes may not be accurately extrapolated from in vitro results. The known concentrations of botanical supplements have been used to assess their influence on target proteins or gene expression, however cellular level in vivo findings are unavailable. Thus, it is difficult to investigate the interaction between botanical supplements and drugs. Therefore, regardless of interaction between botanical supplements and drugs, only retrospective investigations are being performed at present [2].

## 6. Selected Botanical Supplements and their Interactions

A wide array of studies on in vitro and in vivo interactions between various botanicals and transporters are available in literature. Herb-drug interactions were reviewed few years ago [37,38], however many recent studies have added new knowledge to this subject and are reviewed here. This article mainly discusses 10 extensively used botanical supplements that exhibit profound pharmacokinetic interactions with concomitantly used drugs. The in vitro studies revealing the influence of these botanical supplements on various transporters are summarized in Table 1. Moreover, Table 2 describes the interactions of these botanical supplements with different concomitantly used drugs.

### 6.1. Citrus paradise (Grapefruit) Juice

A wide array of studies has reported the interaction of grapefruit juice with drugs. The first study of this kind, conducted in individuals receiving felodipine together with grapefruit juice, documented a significant increase in its AUC with increased heart rate and lowered blood pressure, likely owing to an increase in its bioavailability [72]. Moderate to severe side effects are observed when grapefruit juice interacts with drugs sharing CYP3A4 as their metabolizing enzyme [98,99]. The examples of drugs metabolized by CYP3A4 are β-blockers, antimalarials and calcium channel blockers.

Moreover, at low concentrations, grapefruit juice modulates P-gp activation in vitro, while P-gp are inhibited at high concentrations of this juice, revealing concentration-dependent biphasic transporter modulation by grapefruit juice. In fact, grapefruit juice enhances the transport of vinblastine and talinolol across Caco-2 cells, showing the suppressive role of P-gp [100,101]. Another study conducted on Madin-Darby canine kidney (MDCK)/MDR cell monolayers treated with low concentrations (<5% *v*/*v*) of grapefruit juice, showed significant reduction in the basolateral-to-apical transport of vinblastine across cell layers [102]. In addition, grapefruit juice doubled the rat plasma levels of talinolol, showing grapefruit juice-mediated inhibition of P-gp [103], however, grapefruit juice neither influenced plasma levels of digoxin in human, nor affected the gut P-gp-contents [71,104]. On the other hand, contradictory results were observed whereby grapefruit juice induced a significant reduction in the bioavailability of fexofenadine and celiprolol, possibly due to suppression of uptake transporters by grapefruit juice rather than affecting P-gp and CYP3A4 [44,69]. 

In fact, grapefruit juice-mediated inhibitory effect on OATP1A2 and OATP2B1 has been confirmed in vitro and in vivo for aliskiren, celiprolol, fexofenadine, repaglinide and talinolol [105]. Other OATP substrates are being studied now to assess the relevance of grapefruit juice interactions with other drugs through OATPs.

Naringenin is the most prevalent flavonoid found in grapefruit juice and has strong OATP1A2 inhibition effects. On the other hand, OATP2B1-mediated transport of estrone-3-sulfate is inhibited in vitro by naringen and quercetin which are two main inhibitors of OATP2B1 in grapefruit juice having very low IC_50_ values (in a micromolar range). Grapefruit juice contains some other flavonoids including kaempferol, hesperidin, naringenin and phloretin, which have strong OATP2B1 inhibition activity [105,106].

So far, P-gp inhibition effect of grapefruit juice ingredients has been described in few publications, while cellular studies have demonstrated the decline in P-gp-mediated efflux of vincristine and saquinavir in the presence of naringenin and bergamottin. In addition, at low concentrations, quercetin and kaempferol modulates P-gp activation in vitro, while P-gp are inhibited at high concentrations, revealing concentration-dependent biphasic transporter modulation by these two flavonoids [107,108]. On the basis of existing clinical studies, in the future “this drug may interact with grapefruit juice” labelling may be required by the FDA for certain drugs such as felodipine, cyclosporine and simvastatin.

### 6.2. Citrus sinensis (Orange) Juice

Keeping in view the interactions mediated by grapefruit juice, other citrus fruits have also been screened for any potential inhibitory effects on concomitantly administered drugs. In comparison to sweet oranges, bitter oranges have significantly higher potential to suppress CYP3A4. An important reason for this difference is the presence of bergamottin and its metabolite (6′,7′-dihydroxy-bergamottin) in bitter oranges [108]. A study has reported 76% higher value of plasma felodipine concentration when coadministered with bitter orange juice as compared with sweet orange juice [109]. Since sweet orange juice shows no effect on CYP3A4, clinically significant interactions have been exhibited by this juice, mediated through OATPs. Its interactions were found more significant than those for grapefruit juice. The plasma levels of various OATP2B1 substrates such as atenolol and fexofenadine were markedly decreased in healthy volunteers when cotreated with sweet orange juice. In fact, orange juice-mediated inhibition of OATP2B1 is demonstrated by in vitro studies [65,110], the screening of sweet orange juice interactions mediated through OATP are necessary in human.

### 6.3. Malus pumila (Apple) Juice

To date, the role of CYP3A4 and P-gp in the interactions of apple juice with drugs in human has not been investigated. However, several studies suggest that apple juice interacts significantly with OATP2B1. The coadministration of apple juice and fexofenadine exhibit significant decline in plasma drug concentration, which are more significant than those noted for sweet orange juice and grapefruit juice [44]. A previous study has already demonstrated the inhibitory effect of apple juice (about 250 mL) on OATP2B1, revealing that large volumes or repeated intake of apple juice is not required for OATP2B1. Due to this reason, OATP2B1 expressed in *Xenopus laevis* oocytes is inhibited by apple juice for longer period of time than that by grapefruit juice [110]. Moreover, the bioavailability of several OATP2B1 substrates such as fexofenadine, atenolol and talinolol was significantly decreased by apple juice in human subjects [111]. It is remarkable that concurrently administered apple juice and grapefruit juices did not affect the bioavailability of OATP2B1 substrates such as pravastatin and pitavastatin [105]. It indicates that their absorption depends on OATP2B1 to an insignificant extent. It can also be justified on the basis of fact that diminished intestinal uptake is moderated by decreased uptake into the liver cells. So far, no study has reported the elucidation of the apple juice compounds that induce OATP inhibition in the intestine, although OATP2B1 is significantly inhibited by phloridzin, a flavonoid present in apple juice [64,65,106]. It is remarkable that the interactions of fruit juices with drugs cannot be anticipated precisely due to variable concentrations of apple juice constituents responsible for OATP inhibition depending on fruit species, harvesting season, extraction and storage conditions.

### 6.4. Silybum marianum (Milk Thistle)

Upper alimentary canal and liver diseases are traditionally treated by many natural remedies including *Silybum marianum*, generally known as milk thistle [112]. Milk thistle seeds contain several constituents, which are extracted for further use. The term “silymarin” is generally used for milk thistle seed extract [113]. Silymarin contains silybin (also named as silibinin) as key ingredient, which is a flavonolignans mixture; however, small quantities of chemically unknown constituents are also present in silymarin [114]. Although the clinical effectiveness of silymarin has been reported in several publications, no study evidently describes the clinical efficacy of thistle milk products [115,116]. This scarcity could be attributed to the ambiguous terminologies used for thistle milk products [114]. The intoxications due to ingestion of *Amanita phalloides*, a lethal fungus, has been effectively treated by silymarin [114,116,117], while a formulation with defined quantities of its constituents is being marketed [117]. The literature study showed no evidence about the accurate mode of silymarin action against *Amanita phalloides*, but the antioxidant potential, among others, is an important property of this botanical supplement [112,117,118].

Amatoxins (amanitin) and phallotoxins (phalloidin) are the major toxins in *Amanita phalloides* [117]. These toxins are transported via OATPs in human hepatocytes: OATP1B3 and OATP1B1 mediate the transport of amanitin [119] and phalloidin, respectively [120,121]. Moreover, rat NTCP also mediate the transport of amanitin [119]; however, in this context, the role of human NTCP has not been studied yet. It is remarkable that OATP1B3 is inhibited by silibinin dihemisuccinate, which can thus suppress the transport of amanitin [118,119]. It is concluded that the intoxications due to ingestion of *Amanita phalloides* is likely cured by silibinin dihemisuccinate via suppression of amanitin uptake into hepatocytes.

In case of failure of standard therapy in curing hepatitis C infection, patients have been successfully treated with high-dose of intravenously administered silibinin dihemisuccinate [122,123,124,125]. Moreover, this regimen helps in treating patients with hepatitis C and human immunodeficiency virus (HIV) coinfections [126]. Hepatitis C patients treated with high-dose silibinin dihemisuccinate have the elevated levels of bilirubin [61]. OATP1B3 and OATP2B1 are competitively inhibited in vitro by silibinin and its *Ki* values are similar to those observed for plasma levels of silibinin dihemisuccinate. On the other hand, studies have revealed complex interactions between silibinin dihemisuccinate and OATP1B1: silibinin dihemisuccinate suppresses the high-affinity binding site (IC_50_ = 3.8 μM), while silibinin stimulates the transport by low-affinity binding site at a concentration <10 μM but suppresses at higher concentration [61]. Additionally, bilirubin transport is mediated by OATP1B1 and OATP1B3 [127]. Moreover, MRP2 is inhibited by silibinin dihemisuccinate, which has no interaction with BSEP and NTCP [61].

### 6.5. Camellia sinensis Leaves (Green Tea)

Green tea is used worldwide. Different dietary supplements and beverages are prepared from green tea extracts. Various in vitro and in vivo studies involving human intestinal and hepatic microsomes have revealed the inhibitory effect of green tea on CYP3A4 [105], resulting in the significantly increased AUC of simvastatin compared with control group [128]. So far, the available studies on the interaction between green tea and CYP3A4 substrates have not reported any clinically profound findings. In a four week study, the daily intake of green tea catechins at a dose having 800 mg epigallocatechin gallate (EGCG), subsequently buspirone bioavailability was increased by 20%, likely due to CYP3A4 inhibition [82]. Moreover, the in vitro and animal studies on green tea catechins show that P-gp is not affected by epicatechin, whereas EGCG, epicatechin gallate (ECG) and epigallocatechin have modulating effect on P-gp, with the highest potency for EGCG and the lowest for epigallocatechin (EGC) [53]. However, so far no study describe the determination of the clinical significance of P-gp substrate inhibition by green tea catechins. Another study described the estrone-3-sulfate uptake inhibition by the green tea catechins EGCG and ECG in the cells expressing OATP1A2, OATP1B1 and OATP2B1 [62]. It is also found in cytotoxicity studies that EGCG is transported by OATO1B3 [15]. Remarkably, the average concentration of EGCG and ECG in the brewed green tea was approximately 400 μM, so that the maximal concentrations were in the low millimolar range [62]. Therefore, an intestinal concentration of EGCG and ECG in the same range of the IC_50_ values may be achieved by taking a few cups of green tea, and as a result, clinically significant interactions may be observed. In fact, a clinical study showed significant decline in the AUC of nadolol after repeated use of a catechol rich green tea [83]. Future studies may be focused on the interaction of catechins-enriched green tea supplements with OATP1A2 and OATP 2B1.

### 6.6. Glycine max Merrill (Soybean)

The menopausal women regularly use soy extracts or its pure compounds to compensate harmonic regulations. The major isoflavones in soy are genistein and daidzein, which have low oral bioavailability due to their extensive glucuronidation in the intestine and liver. MRP2 extrudes glucuronides into bile, thus activating bile flow by approximately 15% in isolated perfused rat liver [129]. So far, no data is available on the choleretic effect in humans after oral administration of soy supplements. However, genistein may competitively inhibit MRP2-mediated biliary excretion of glucuronides, and same finding was observed when genistein addition diminished biliary excretion of bilirubin conjugates by 76% in a rat model [129]. Genistein and daidzein, both are known as ABCG2 inhibitors, profoundly inhibited milk secretion of ABCG2 substrate danofloxacin, resulting in about 50% decline in its milk/plasma ratio [42]. The role of genistein as P-gp inducer and OATP1B1 inhibitor has been demonstrated using various cellular models [48,63], in vivo data in this context is not available. However, two studies in human suggested significant drug interactions of soy milk on warfarin deficiency, resulting in reversible subtherapeutic international normalized ratio (INR), enhancing thromboembolic risk [84,130]. Further studies are required to investigate whether soy milk-induced INR decrease is triggered by changes in warfarin transport, its metabolism or both. The coadministration of warfarin with soy supplements needs essential care.

### 6.7. Hypericum perforatum (St. John’s Wort)

Above discussion outlines the inhibition of transporters by botanical supplements. Besides, the regulation of gene expression is affected by the supplements, for instance, *Hypericum perforatum* (*H. perforatum*, St. John’s wort). Placebo-controlled clinical trials have proved that the effectiveness of *H. perforatum* in depression is comparable to standard therapies [131]. Hypericin is one of the major constituents of *H. perforatum*; however, *H. perforatum* contains other constituents, which contribute to the hypericin action [132]. Several in vitro and animal studies have been conducted to explore the mode of action of *H. perforatum* [133,134]. *H. perforatum* extracts interact with several drugs, likely due to complex composition of this supplement [135,136]. Hyperforin is the major ingredient that contribute to these interactions as well as activates the nuclear transcription factor pregnane X receptor [137]. Moreover, several CYPs are inhibited by crude extracts of *H. perforatum* [138]. These two results reveal that the inconstant composition of *H. perforatum* extracts affects drug disposition that is difficult to be determined in individual formulations.

The studies have reported the extremely low bioavailability of cyclosporine in transplant patients using *H. perforatum* extracts [139,140,141]. The impact of *H. perforatum* extract on digoxin bioavailability in both human and rats was investigated at the protein level [45]. This study described the enhanced induction of hepatic rCYP3A2 by 2.5-fold and intestinal P-gp by 3.8-fold in rats supplemented with *H. perforatum* extract for 14 days [45]. On the other hand, the expression of intestinal rCYP3A2 was enhanced by 2.5-fold and hepatic P-gp was unaffected. Moreover, the increased induction of intestinal CYP3A4 by 1.5-fold and P-gp by 1.4-fold in humans supplemented with *H. perforatum* extract for 14 consecutive days was also reported [45]. *H. perforatum* extract intake effects on human-induced hepatic CYP3A4 were evidenced by increased demethylation of erythromycin. In parallel with these changes, the AUC_0–7h_ of digoxin was also reduced. Hence, uptake of a reduced P-gp substrate (for example, cyclosporine) across the apical membrane of enterocytes and an increased metabolism of CYP3A4 substrates by *H. perforatum* was described, explaining that reduced plasma levels of cyclosporine are likely due to an increased first pass effect. These findings clarify the effects of *H. perforatum* on the pharmacokinetics and thus the pharmacodynamics of several drugs and indicate the compromised efficacy of oral contraceptives [142,143]. The above study on *H. perforatum* evidently describes the moderate level botanical supplement-mediated change in expression levels of key enzymes and transporters involved in the first-pass effect, influencing the pharmacokinetics that may lead to therapeutic failure of drugs with severe clinical consequences [144,145]. Therefore, it is critically important that the health-care professionals counsel and educate the patients about the use of botanical supplements.

### 6.8. Cynara scolymus (Artichoke)

The traditional use of botanical products for human health care started several thousands of years ago [146,147]. People across the world generally use botanical supplements to treat hepatic disorders, including among others hepatitis C [148,149]. The ancient Greeks used *Cynara scolymus* (*C. scolymus*; artichoke) leaf extracts, which are useful for choleretic and anticholestatic effects and to reduce lipid contents [150,151]. The induction of choleresis in rats has already been demonstrated [152]. The choleretic effect is also observed in human, as evident from the augmented release of bile into duodenum in humans supplemented with *C. scolymus* [153]. Cynarin and luteolin are major ingredients of *C. scolymus* extracts [154]. Cynarin weakly inhibits OATP2B1 [155], and thus is capable of reducing OATP2B1-mediated uptake of drugs by the liver and intestinal cells. However, no evidence is available on whether cynarin is an OATP2B1 substrate. Moreover, the literature study reveals no description of luteolin transporters, although the metabolism of luteolin widely produces glucuronides that may act as ABCG2 substrate [156].

### 6.9. Resveratrol

Resveratrol is a stilbenoid and belongs to the polyphenol group of natural compounds. Resveratrol can be isolated in low quantities from red grapes, red wine and berries [157]. A large number of preclinical and clinical studies have demonstrated the antiinflammatory, antiobesity, antidiabetic, antiaging, anticancer and neuroprotective role of resveratrol [158]. Resveratrol is extensively metabolized into sulfates and glucuronides in the intestine and liver, resulting in low concentrations of intact resveratrol in blood and tissues. As a result, the bioavailable levels of resveratrol are inadequate to explain its observed pharmacological activities [159,160]. Therefore, since resveratrol is categorized as a dietary supplement, the USA Food and Drug Administration (FDA) has not approved its efficacy as a drug.

The active transport of resveratrol and its conjugates has been demonstrated by many in vitro and animal studies. Whereas the efflux of resveratrol glucuronides occurs through MRP2 and MRP3 [161,162]. Moreover, the kinetics of resveratrol uptake by OATP1B1, OATP1B3 and OATP2B1 is a saturable process. Only OATP1B3 transports resveratrol-3-*O*-sulfate, while both OATP1B1 and OATP1B3 are involved in resveratrol-3-*O*-4′-*O*-disulfate transportation. It is remarkable that none of the three OATPs have affinity with resveratrol-4′-*O*-sulfate, resveratrol-3-*O*-glucuronide and resveratrol-4′-*O*-glucuronide [16].

The safety profiles of resveratrol have suggested it a safe remedy having no undesired effects on human health if taken alone in a concentration less than 100 mg per day, even for several months. However, higher doses of resveratrol (≥1 g) can produce some undesired effects, such as gastrointestinal problems and hot flashes.

ABC transporters are inhibited in vitro by resveratrol, for instance, resveratrol ameliorates the cytotoxicity of doxorubicin and docetaxel through the suppression of P-gp and downregulation of the *ABCB1* gene [163]. Similarly, resveratrol enhances the intestinal absorption of bestatin via downregulation of *ABCB1* and *ABCC2* gene and their respective mRNA levels [164]. Another study in non-small-cell lung cancer cell lines has revealed resveratrol-mediated inhibition of ABCG2 [165]. However, the evidences for resveratrol-mediated inhibition of ABC transporters and OATPs in patients and the subsequent drug interactions are scarce at best. In addition, same studies may be carried out for resveratrol metabolites. Resveratrol-transporter interaction may be tested only in the intestine, since 8 day treatment of colon cancer patients with resveratrol (1 g/day) achieved high concentrations for resveratrol (674 μM) and its metabolite resveratrol-3-*O*-sulfate (67 μM) [166]. The concentration of resveratrol-3-*O*-4′-*O*-disulfate can be presumed to be equal to resveratrol-3-*O*-sulfate, since both metabolites are currently identified at the same level in human plasma [167]. Resveratrol-containing supplements must be taken cautiously, since several supplements have more than 250 mg of resveratrol. In fact, the interaction of resveratrol with drugs is supported by many earlier studies. For instance, coadministration of resveratrol (1 g/day) with cytochrome P450 (CYP) substrates to healthy individuals showed the inhibition of CYP2C9, CYP2D6 and CYP3A4, resulting in increased side effects [168]. Actually, resveratrol (500 mg/day as single dose for 10 day) coadministered with diclofenac and carbamazepine to healthy individuals showed significant increases in maximum plasma concentration (C_max_) and area under zero moment curve (AUC) of both drugs. This increase in pharmacokinetic parameters was attributed to the inhibition of CYP2C9 and CYP3A4, respectively.

### 6.10. Anthocyans

Anthocyans occur in plants as flavonoid pigments that contribute the purple, blue, and red colors of grapes and berries. Anthocyans include both glycodies and aglycones, termed as anthocyanins and anthocyanidins, respectively. Anthocyanin-enriched water extracts and juices are available as dietary supplements to achieve various health benefits. It is remarkable that various dietary supplements contain high concentrations (approximately 200 mg) of anthocyanins per dose. Anthocyanins and anthocyanidins have inhibitory effect on various CYP isozymes in vitro. For instance, delphinidin and pelargonin have CYP3A4 and CYP2C9 inhibitory effects, respectively [169]. In addition, P-gp and ABCG2 are slightly inhibited by berry anthocyanins [170,171]. However, the confirmation of these in vitro effects needs clinical studies, as while midazolam bioavailability was significantly increased on concurrent use with a double dose of cranberry juice in one study, another study could not confirm this finding [172]. One possible reason of this contradictory finding could be the significant variations in the contents of anthocyanins in various fruits. Cranberry juice has no interaction in human with certain drugs such as tizanidine, amoxicillin and flurbiprofen [172]. The probable interaction between cranberry juice and CYP2C9 substrate warfarin is clinically important. In this context, five clinical studies were conducted that revealed no change in the oral bioavailability [172]. However, the supplementation of cranberry juice concentrate to human followed by single high dose of warfarin resulted in increased INR that was correlated with bleeding episodes [173]. Owing to this reason, the label “warfarin may interact with cranberry juice” is required by the FDA. CYP3A4 and CYP3A9 substrates and large quantities of cranberry juice (more than 1 L per day) or its concentrated supplement (approximately 1 g per day) should not be concurrently used for prolonged time periods. In a previous study, the influence of 21 anthocyanins and their six relevant anthocyanidins on OATP1B3 and OATP1B1 expression in human hepatocytes was investigated in vitro [174]. Pelargonin and dolphin reduced OATP1B3 expression, while OATP1B1 expression was enhanced by malvin, malvidin-3-*O*-galactoside, and cyaniding-3-*O*-sophoroside, revealing that OATP1B3 and OATP1B1 expression can be altered by different anthocyanins. So far, there is no evidence neither of anthocyanin-mediated altered expression of intestinal OATP1B3 and OATP1B1 nor of clinical interaction of anthocyanins with intestinal OATP1B3 and OATP1B1.

### 6.11. Other Botanical Supplements

In addition to the botanical supplements described above, drug disposition might be affected by many other botanical supplements and food ingredients including *Glycyrrhiza glabra* (licorice) [56], *Piper nigrum* (black papper) [47], *Allium sativum* (garlic) [51], *Ginkgo biloba* (ginkgo) [57], *Panax ginseng* (ginseng) [59], *Curcuma longa* (turmeric) [54], *Cimicifuga racemosa* (black cohosh) [66], *Echinacea* family [175] and *Peumus boldo* [46]. It is remarkable that *Allium sativum*, *Echinacea*, *Ginkgo biloba*, *Glycyrrhiza glabra* and *Panax ginseng* are P-gp inhibitors. In rats, P-gp and ABCG2 are induced by piperine and boldine, isolated by *Piper nigrum* and *Peumus boldo*, respectively. Curcumin isolated from *Curcuma longa* inhibit ABCG2, which, in contrast, is induced by diallyl disulphide found in *Allium sativum*. In addition, all these plants and their ingredients have OATP inhibition features. The above findings are obtained from in vitro studies. Only a few of these entities have been investigated for their pharmacokinetic assessment, revealing drug interactions with these botanicals, based on the clinical relevant enzyme- or transporter-based interactions. *Allium sativum* intake resulted in subtherapeutic INR of fluindione due to induction of CYPs [90]. The decline in midazolam AUC was observed on coadministration with *Echinaecea purpurea*, likely due to induction of CYP3A4 in hepatocytes [91], however *Gingko biloba* and *Panax ginseng* inhibited CYP3A4, resulting in increase in AUC of midazolam [94,97]. The ingestion of *Piper nigrum* leads to increase in AUC of phenytoin [176]; however a mechanistic interpretation for this observation is needed.

## 7. Conclusions

The pharmacokinetics and pharmacodynamics of drugs coadministered with botanical supplements are significantly altered. This review article discusses the effects of some botanical supplements on drug transporters in the intestinal and hepatic cells, since the bioavailability of orally administered drugs depends on the first pass effect. The increased first pass effect may lead to subtherapeutic effect, while severe consequences may appear if first pass effect is reduced. To tackle these recognized interaction issues, FDA has requested the addition of warning labels in some instances, such as warfarin. Self-medication with botanical supplements may lead to harmful effects or even the therapeutic failure of drugs, thus our knowledge must be enhanced to avoid unpleasant interactions of botanical supplements with drugs, and hence to improve therapeutic effectiveness. Since plant constituents have a close association with enzymes and transporters for disposition of drugs and xenobiotics in human and animals, the understanding of drug disposition can be enhanced through improved understanding of detailed mechanisms involved in the interaction of botanical supplements and food constituents. Thus, it is also necessary to interpret the mode of interaction between botanical supplements and drugs, however, there are several hindrances in these mechanistic investigations, such as the unavailability of pharmacokinetic data of individual active constituents of botanical supplements in humans, variation in the relative contents of botanical supplements between suppliers and between batches, and interindividual variability of the different transporters and metabolizing enzymes. 

## Figures and Tables

**Table 1:** The selective in vitro studies revealing the role of various botanical supplements in the inhibition/induction of various transporters.

Transporter	Botanicals and Their Effect		References
BCRP	Inhibitor	*Cimicifuga racemosa* extract	[39]
		Curcumin (*Curcuma longa* flavonoid)	[40]
		Kaempferol (*Gingko biloba* flavonoid)	[41]
		Naringenin (Grapefruit flavonoid)	[41]
		Genistein (*Glycine max* flavonoid)	[42]
		*Silybum marianum* extract	[39]
MDR1	Inducer	Garlic extract	[43]
		Grapefruit juice	[44]
		*Hypericum perforatum* extract	[45]
		Bodine (*Peumus boldo* flavonoid)	[46]
		Piperine (*Piper nigrum* flavonoid)	[47]
		Genistein (*Glycine max* flavonoid)	[48]
	Inhibitor	Garlic extract	[49]
		Tangeretin, nobiletin, rutin, allicin (Garlic flavonoid)	[50,51,52]
		epigallocatechin gallate, epicatechin gallate, quercetin (*Camellia sinensis* flavonoid)	[53]
		Curcumin (*Curcuma longa* flavonoid)	[54]
		*Echinacea* extract	[55]
		Glycyrrhetinic acid *(Glycyrrhiza glabra* flavonoid)	[56]
		*Gingko biloba* extract	[57]
		*Hypericum perforatum* extract	[58]
		*Panax ginseng* extract	[59]
MRP1	Inhibitor	Glycyrrhetinic acid *(Glycyrrhiza glabra* flavonoid)	[56]
MRP2	Inducer	Garlic extract	[51]
		Diallyl disulfide (Garlic flavonoid)	[60]
		Bodine (*Peumus boldo* flavonoid)	[46]
		Genistein (*Glycine max* flavonoid)	[48]
	Inhibitor	Curcumin (*Curcuma longa* flavonoid)	[40]
		Silybin A + silybin B (*Silybum marianum* flavonoids)	[61]
OATP1A2	Inhibitor	*Camellia sinensis* extract, epicatechin gallate, epigallocatechin gallate	[62]
		Grapefruit juice	[44]
		Orange juice	[44]
OATP1B1	Inhibitor	Epigallocatechin gallate, epicatechin gallate (*Camellia sinensis* flavonoid)	[62]
		Genistein (*Glycine max* flavonoid)	[63]
		Silybin A + silybin B (*Silybum marianum* flavonoids)	[61]
OATP1B3	Inhibitor	Silybin A + silybin B (*Silybum marianum* flavonoids)	[61]
OATP2B1	Inhibitor	Apple juice	[64]
		Apple juice, phloridzin, phloretin	[64,65]
		*Camellia sinensis* extract, epicatechin gallate, epigallocatechin gallate	[66]
		*Cimicifuga racemosa* extract	[66]
		*Echinacea* extract	[66]
		Kaempferol (*Gingko biloba* flavonoid)	[66]
		Grapefruit juice	[44]
		Orange juice	[44]
		*Glycine max* extract	[66]
		Silybin A + silybin B (*Silybum marianum* flavonoids)	[61]
NTCP	Inhibitor	Apple juice	[44]
BSEP	Inducer	Bodine (*Peumus boldo* flavonoid)	[46]

**Table 2:** Reported interactions between the clinically prescribed drugs and botanical supplements.

Drugs	Botanical Supplements	Effect on Transporters/Enzymes	Pharmacokinetic Change			Refs.
			AUC	C_max_	PR	
Aliskiren	Grapefruit juice	↓ intestinal OATP1A2	↓	↓	-	[67]
Atorvastatin		↓ CYP3A4	-	↑	-	[68]
Celiprolol		↓ OATPs	↓	↓	-	[69]
Cyclosporine		↓ CYP3A4	↑	↑	-	[70]
Felodipine		↓ CYP3A4	↑	↑	↑	[71]
Fexofenadine		↓ intestinal OATP1A2	↓	↓	-	[44]
Nifedipine		↓ CYPs	↑	↑	-	[72]
Saquinavir		↓ CYP3A4	↑	↑	-	[73]
Simvastatin		↓ CYP3A4	↑	↑	-	[74]
Aliskiren	Orange juice	↓ OATP2B1	↓	↓	↓	[75]
Celiprolol		↓ OATPs	↓	↓	-	[76]
Fexofenadine		↓ OATP1A2	↓	↓	-	[44]
Aliskiren	Apple juice	↓ OATP2B1	↓	↓	↓	[75]
Atenolol		↓ OATP2B1	↓	↓	-	[77]
Fexofenadine		↓ OATP1A2	↓	↓	-	[44]
Fexofenadine		↓ OATP2B1	↓	↓	-	[67]
Domperidone	*Silybum marianum*	↓ CYP3A4, ↓ MDR1	↑	↑	-	[78]
Losartan		↓ CYP3A4	↑	↑	-	[79]
Metronidazole		↑ MDR1, ↑ CYP3A4	↓	↓	-	[80]
Talinol		↓ MDR1	↑	↑	-	[81]
Buspirone	*Camelia sinensis*	↓ CYP3A4	↓	-	-	[82]
Nadolol		↓ OATP1A2	↓	↓	↓	[83]
Warfarin	Soybean	↓ OATPs	-	-	↓	[84]
Amitriptyline	*Hypericum perforatum*	↑ MDR1, ↑ CYP3A4	↓	↓	-	[85]
Clozapine		↑ MDR1, ↑ CYPs	-	↓	-	[86]
Cyclosporine		↑ MDR1, ↑ CYP3A4	↓	-	-	[87]
Digoxin		↑ MDR1, ↑ CYP3A4	↓	↓	-	[45]
Fexofenadine (single dose)		↓ MDR1	↑	↑	-	[88]
Fexofenadine (multiple dosing)		↓ MDR1	↓	↓	-	[88]
Indinavir		↑ MDR1, ↑ CYP3A4	↓	-	-	[89]
Fluindione	*Allium sativum*	↑ CYPs	-	-	↓	[90]
Saquinavir		↑ intestinal MDR1	↓	↓		[43]
Midazolam	*Echinacea pupurea*	↑ hepatic CYP3A	↓	-	-	[91]
Alprazolam	*Gingko biloba*	↓ CYP3A4	↓	-	-	[92]
Midazolam		↑ CYP3A	↓	↓	-	[93]
Midazolam		↓ CYP3A4	↑	-	-	[94]
Talinol		↓MDR1	↓	↓	-	[95]
Omeprazole		↑ CYP2C19	↓	-	-	[96]
Midazolam	*Panax ginseng*	↑ CYP3A	↓	↓	-	[97]

AUC: Area under curve, C_max_: maximum plasma drug concentration, PR: pharmacodynamic response, ↓: inhibition/decrease, ↑: induction/increase, -: not reported.
